# Host Cell Glycocalyx Remodeling Reveals SARS-CoV-2 Spike Protein Glycomic Binding Sites

**DOI:** 10.3389/fmolb.2022.799703

**Published:** 2022-03-14

**Authors:** Ying Sheng, Anita Vinjamuri, Michael Russelle S. Alvarez, Yixuan Xie, Marisa McGrath, Siyu Chen, Mariana Barboza, Matthew Frieman, Carlito B. Lebrilla

**Affiliations:** ^1^ Department of Chemistry, University of California, Davis, Davis, CA, United States; ^2^ The Biochemistry, Molecular, Cellular and Developmental Biology (BMCDB) Graduate Group, University of California, Davis, Davis, CA, United States; ^3^ University of the Philippines Los Baños, Los Baños, Philippines; ^4^ Department of Microbiology and Immunology, University of Maryland School of Medicine, Baltimore, MD, United States; ^5^ Department of Anatomy, Physiology and Cell Biology, School of Veterinary Medicine, University of California, Davis, Davis, CA, United States

**Keywords:** SARS-CoV2, glycocalyx, LC/MS, remodeling glycome, host-virus interaction, spike (S) protein, ACE2 glycosylation

## Abstract

Glycans on the host cell membrane and viral proteins play critical roles in pathogenesis. Highly glycosylated epithelial cells represent the primary boundary separating embedded host tissues from pathogens within the respiratory and intestinal tracts. SARS-CoV-2, the causative agent for the COVID-19 pandemic, reaches into the respiratory tract. We found purified human milk oligosaccharides (HMOs) inhibited the viral binding on cells. Spike (S) protein receptor binding domain (RBD) binding to host cells were partly blocked by co-incubation with exogenous HMOs, most by 2-6-sialyl-lactose (6′SL), supporting the notion that HMOs can function as decoys in defense against SARS-Cov2. To investigate the effect of host cell glycocalyx on viral adherence, we metabolically modified and confirmed with glycomic methods the cell surface glycome to enrich specific N-glycan types including those containing sialic acids, fucose, mannose, and terminal galactose. Additionally, Immunofluorescence studies demonstrated that the S protein preferentially binds to terminal sialic acids with α-(2,6)-linkages. Furthermore, site-specific glycosylation of S protein RBD and its human receptor ACE2 were characterized using LC-MS/MS. We then performed molecular dynamics calculations on the interaction complex to further explore the interactive complex between ACE2 and the S protein. The results showed that hydrogen bonds mediated the interactions between ACE2 glycans and S protein with desialylated glycans forming significantly fewer hydrogen bonds. These results supported a mechanism where the virus binds initially to glycans on host cells preferring α-(2,6)-sialic acids and finds ACE2 and with the proper orientation infects the cell.

## Introduction

Severe acute respiratory syndrome coronavirus-2 (SARS-CoV-2), the causative agent of COVID-19 ((Zhu et al., 2020)), encodes an extensively glycosylated spike (S) protein that protrudes from the viral surface and binds angiotensin-converting enzyme 2 (ACE2) on host cells ((Hoffmann et al., 2020; Walls et al., 2020; Wan et al., 2020; Wrapp et al., 2020; Gstöttner et al., 2021)). This novel SARS-CoV-2 was found to share similarities with the SARS-CoV, which was responsible for the SARS pandemic that occurred in 2002 ((Peiris et al., 2003; Viruses, 2020)). ACE2 serves as the entry point for several coronaviruses into cells, including SARS-CoV and SARS-CoV-2 ((Li et al., 2003; Letko et al., 2020)). The receptor binding domain (RBD) of SARS-CoV-2 S protein has been limited to amino acid residues Arg319 to Phe541 ((Lan et al., 2020; Tai et al., 2020; Tian et al., 2020)). *In vitro* binding measurements also showed that the SARS-CoV-2 RBD binds to ACE2 with an affinity in the low nanomolar range, indicating that the RBD is a key functional component within the S1 subunit responsible for the binding of SARS-CoV-2 to ACE2 ((Tian et al., 2020; Walls et al., 2020)). The plasma membrane protein ACE2 is abundantly expressed in humans tissues, including respiratory and intestinal epithelia, liver arteries, heart and kidney ((Hamming et al., 2004)).

Mammalian epithelial cells are highly glycosylated ((Park et al., 2015; Park et al., 2017)) due to glycoproteins and glycolipids found on the cell membrane. Both the ACE2 receptor and the S protein are similarly extensively glycosylated. Several glycosylation sites are found near the binding interface ((Park et al., 2015; Park et al., 2017), (Shajahan et al., 2020; Watanabe et al., 2020; Shajahan et al., 2021)). The role of glycosylation in the interaction between human ACE2 and SARS-CoV-2 S protein has been extensively studied, primarily using molecular dynamics (MD) simulations ((Shajahan et al., 2020; Watanabe et al., 2020; Shajahan et al., 2021), (Zhao et al., 2020; Mehdipour and Hummer, 2021)). Human ACE2 variants have also been modeled, characterized, and examined for susceptibility to coronavirus interactions ((Chan et al., 2020; Eric et al., 2021)). Among ACE2 glycosylation sites, one of the most characterized position for its role in S protein binding and viral infectivity is the asparagine on position 90 (N90). Recent genetic and biochemical studies showed that mutations that removed glycosylation on N90 site directly increased the susceptibility to SARS-CoV-2 infection ((Chan et al., 2020; Zhao et al., 2020)). In contrast, glycans present on N322 and N90 have the opposite effects on S protein binding. The N322 glycan interacts tightly with the RBD of the ACE2-bound S protein and strengthens the complex ((Mehdipour and Hummer, 2021)). The S protein also contains glycosaminoglycan (GAG) binding motifs so that host surface GAGs contribute to cell entry by SARS-CoV-2 (Kim et al., 2020). Additionally, heparan sulfate has also been shown to promote spike-ACE2 interaction (Clausen et al., 2020).

Pathogen adhesion is often mediated by highly specific lectin-glycan interactions. For example, *Escherichia coli* with type 1 fimbriae binds to cell surfaces exhibiting preference for high mannose glycans, while *Escherichia coli* with type S fimbriae has binding specificity for α-(2,3)-linked sialic acids. Cell surface glycans have also been shown to act as a shield to mask its identity as a viable host to the pathogen. It was recently proposed that HMOs can prevent viral adhesion to intestinal epithelial cells *via* binding to the epithelial surface, causing structural changes in the receptor thereby impeding the virus from hijacking the host cell ((Moore et al., 2021)). Breast-fed infants have significant amounts of HMOs lining the mucosal surface of their gastrointestinal tract. While the viral binding to glycans and HMO in particular have been studied, the direct interaction between the virus and host glycans remain relatively unexplored.

In this study, the role of host glycosylation and its effect on S protein binding was examined by identifying the host glycans that are involved in the binding. The study began with HMOs in a rapid assay to determine the broad details of the oligosaccharide that bind the virus. We then examined the impact of host cell glycosylation on S protein binding, by modifying the host glycosylation while leaving protein expression unchanged using transferase inhibitors. Using newly developed methods glycomic tools, we found that specific glycans on the host cell facilitate S protein binding and that binding depends more on the nature of glycans than it does on the membrane proteins.

## Methods and Materials

### HMO Purification

HMOs were obtained from breast milk samples using previously reported methods ((Wu et al., 2010; Wu et al., 2011)). Briefly, breast milk samples from seven mothers were pooled. Pooled sample was defatted through centrifugation, proteins were precipitated with ethanol, and the resulting glycans were reduced with sodium borohydride (Sigma-Aldrich, St. Louis, MO, United States). Solid phase extraction was performed on 25 mg graphitized carbon cartridges (ThermoFisher). Solvents were dried in vacuo using miVac (SP Scientific, PA, United States) and purified HMOs were reconstituted and diluted prior to analysis. The individual HMO compounds 2′-fucosyllactose (GKAD-02001; Agilent), 6′- sialyllactose (GKAD-02013; Agilent), and lacto-N-neotetraose (GKAD-02005; Agilent), were tested in their native state.

### Inhibition of HMO Against SARS-CoV-2

All HMO screens were performed with Vero E6 cells. Cells were plated in 96 well plates at 5e3 cells/well one day prior to infection. HMOs were diluted from stock to 50 μM and an 8-point 1:2 dilution series was prepared in duplicate in Vero Media. Every compound dilution and control were normalized to contain the same concentration of HMO vehicle (e.g., DMSO). Cell plates were pre-treated with the HMO for 2 h at 37°C (5% CO_2_) prior to infection with diluted SARS-CoV-2 GFP for a final MOI of 0.1. In addition to plates that were infected, parallel plates were left uninfected to monitor cytotoxicity of HMO alone, measured by CellTiter-Glo (CTG) assays as per the manufacturer’s instructions (Promega, Madison, WI, United States). Plates were then incubated at 37°C (5% CO_2_) for 48 h, followed by fixation with 4.0% paraformaldehyde, nuclear staining with Hoechst (Invitrogen, Carlsbad, CA, United States), and data acquisition on a Celigo 5-channel Imaging Cytometer (Nexcelom Bioscience, Lawrence, MA, United States). The percent of infected cells was determined for each well based on GFP expression by manual gating using the Celigo software. For the CTG assays, luminescence was read on a BioTek Synergy HTX plate reader (BioTek Instruments Inc., Winooski, VT, United States) using the Gen5 software (v7.07; Biotek Instruments Inc., Winooski, VT, United States).

### Cell Culture and Glycocalyx Remodeling Treatments

Human liver hepatocellular carcinoma HepG2, lung carcinoma epithelial Calu-3, urinary bladder epithelial RT4 cells were obtained from American Type Culture Collection (ATCC, VA, United States). HepG2 and Calu-3 cells were grown in Eagle’s Minimum Essential Medium (EMEM). RT4 cells were cultured in McCoy’s 5a Medium. All media were supplemented with 10% (v/v) fetal bovine serum and 100 U ml^−^1 penicillin and streptomycin. Cells were subcultured at 90% confluency and maintained at 37°C in a humidified incubator with 5% CO_2_. At 50% cell confluency, the cells were either treated with 150 μM kifunensine, 2-fluoro-L-fucose, or 3-fluorinated sialic acid for 48 h.

### Sample Information

Recombinant human angiotensin-converting enzyme 2 (ACE2) (RayBiotech, Georgia, Product Number 230-30165), SARS-CoV-2 spike protein S1 Subunit RBD (Arg319-Phe541) (RayBiotech, Georgia, Product Number 230-30162) and spike protein S1 subunit (Val16-Arg685) (Sino Biological, China, Product Number 40591-V08H) were all derived from transfected human HEK293 cells. The recombinant proteins had C-terminal His-tags but were not conjugated to the fluorophore.

### Immunofluorescence

The cells were seeded into FluoroDish^™^ cell culture dishes (WPI, FL) coated with poly-d-lysine with appropriate density using EMEM cell culture media. At 40% confluency, cells were treated with media either supplemented with 150 μM kifunensine, 2-fluoro-L-fucose, or 3-fluorinated sialic acid for 48 h. Control cell culture without treatment and treated cells were rinsed with phosphate-buffered saline (PBS), and fixed with 4% paraformaldehyde (Affymetrix, OH). Recombinant SARS-CoV-2 spike protein RBD and S1 subunits were conjugated to a fluorescent label with Alexa Fluor^™^ 555 according to manufacturing instructions (Microscale Protein Labeling Kit, Invitrogen, MA, United States). Fixed control and glyco-modified cells were then incubated with fluorescent labelled S proteins or Anti-ACE2 antibody (Santa Cruz Biotechnology, TX, United States) in PBS at 4°C for 18 h. Cells were stained for the nucleus with 1.6 μM Hoechst 33342 (Thermo Fisher Scientific, MA, United States) followed by the staining for the plasma membrane with 1000-fold diluted CellMask^™^ Deep Red Plasma Membrane Stain (Thermo Fisher Scientific, MA, United States), respectively at 37°C for 10 min. Fluorescence images were captured using a Leica TCS SP8 STED 3X Super-Resolution Confocal Microscope (Wetzlar, Germany). Fluorescence intensity was quantified for selected cell area. Quantification was performed with software ImageJ.

### Cell Membrane Extraction

Cell membrane fractions were prepared as previously described (Park et al., 2015; Li et al., 2019; Li et al., 2020). Briefly, control and glycoengineered cells were collected and resuspended in homogenization buffer containing 0.25 M sucrose, 20 mM HEPES-KOH (pH 7.4), and protease inhibitor mixture (1:100; Calbiochem/EMD Chemicals). Cells were lysed on coolrack (Corning, MA, United States) with five alternating on and off pulses in 5 and 10 s intervals using a probe sonicator (Qsonica, CT, United States). Nuclear and mitochondrial fractions and cellular debris were pelleted by centrifugation at 2000 × *g* for 10 min. The supernatants were then submitted for ultra-centrifugation at 200,000 × *g* for 45 min at 4°C to extract the plasma membrane.

### Enzymatic N-Glycan Release and Purification of N-Glycans

Details of the glycomic sample preparation have been described previously ((Wu et al., 2010; Wu et al., 2011)). Extracted cell membrane fractions or protein (RNase B) were suspended with 100 μl of 100 mM NH_4_HCO_3_ in 5 mM dithiothreitol and heated in boiling water for 2 min to denature the proteins. Solutions of with 2 μl of peptide N-glycosidase F (New England Biolabs, MA, United States) were added to the samples, and the resulting solutions were then incubated in a microwave reactor (CEM Corporation, NC, United States) at 20 W, 37°C for 10 min. The samples were further placed in a 37°C water bath for 18 h. Ultracentrifugation at 200,000 × *g* for 45 min was performed to precipitate proteins, and the supernatant containing N-glycans was collected and desalted using porous graphitic carbon (PGC) on a 96-well SPE plate (Grace, IL, United States). The plate was equilibrated with 80% (v/v) acetonitrile containing 0.1% (v/v) trifluoroacetic acid. Then the samples were loaded onto the plate and washed with nanopure water. N-Glycans were eluted with a solution of 40% (v/v) acetonitrile containing 0.05% (v/v) trifluoroacetic acid, and dried in vacuo using miVac (SP Scientific, PA, United States) prior to further analysis.

### Glycoprotein Digestion and Enrichment

Details of the protein digestion have been described previously ((Wu et al., 2010; Wu et al., 2011)). Extracted cell membrane proteins were reconstituted in 60 μl of 8 M urea. Recombinant proteins and dissolved cell membrane proteins were reduced with 2 μl of 550 mM dithiothreitol, and then alkylated with 4 μl of 450 mM iodoacetamide. A 420 μl of 50 mM ammonium bicarbonate solution was added to dilute the urea concentration and to adjust the pH value. The samples were incubated with trypsin at 37°C for 18 h. The resulting peptides were concentrated *in vacuo* using miVac (SP Scientific, PA, United States). Glycopeptides were enriched by solid-phase extraction using iSPE^®^-HILIC cartridges (HILICON, Sweden). The cartridges were conditioned with 0.1% (v/v) trifluoroacetic acid in acetonitrile, followed by 1% (v/v) trifluoroacetic acid and 80% (v/v) acetonitrile in water. The samples were loaded and washed with 1% (v/v) trifluoroacetic acid and 80% (v/v) acetonitrile in water. The enriched glycopeptides were eluted with water containing 0.1% (v/v) trifluoroacetic acid and dried prior to mass spectrometric analysis.

### Glycomic Analysis With LC-MS/MS

Details of the glycomic MS analysis have been described previously ((Wu et al., 2010; Wu et al., 2011)). Glycan samples were reconstituted with 30 μl nanopure water and analyzed using an Agilent 6520 Accurate Mass Q-TOF LC/MS equipped with a PGC nano-chip (Agilent Technologies, CA, United States). The glycan separation was performed at a constant flow rate of 300 nl min −1, and a binary gradient was applied using (A) 0.1% (v/v) formic acid in 3% acetonitrile and (B) 1% (v/v) formic acid in 90% acetonitrile: 0–2 min, 0–0% (B); 2–20 min, 0–16% (B); 20–40 min, 16%–72% (B); 40–42 min, 72–100% (B); 42–52 min, 100–100% (B); 52–54 min, 100–0% (B); 54–65 min, 0–0% (B). MS spectra within the mass range of m/z 600–2000 were collected at a rate of 1.5 s per spectrum in positive ionization mode. The most abundant precursor ions in each MS1 spectrum were subjected to fragmentation through collision-induced dissociation (CID) based on the equation V collision = 1.8 × (m/z)/100–2.4 V.

### Glycomic Data Analysis

Details of the glycomic data analysis have been described previously ((Wu et al., 2010; Wu et al., 2011)). Extraction of the compound chromatographs of glycans from cells was obtained *via* the MassHunter Qualitative Analysis B08 software (Agilent, CA, United States). N-Glycan compositions were identified according to accurate masses using an in-house library constructed based on the knowledge of N-glycan biosynthetic pathways and previously obtained in-house structures of N-glycans. Relative abundances were determined by integrating peak areas for observed glycan masses and normalizing to the summed peak areas of all glycans detected.

### Glycoproteomic Analysis With LC-MS/MS

Details of this analysis have been described previously ((Wu et al., 2010; Wu et al., 2011)). The enriched glycopeptide samples were reconstituted with nanopure water and directly characterized using UltiMate^™^ WPS-3000RS nanoLC 980 system coupled to the Nanospray Flex ion source of an Orbitrap Fusion Lumos Tribrid Mass Spectrometer system (Thermo Fisher Scientific, MA, United States). The analytes were separated on an Acclaim^™^ PepMap^™^ 100 C18 LC Column (3 μm, 0.075 mm × 150 mm, ThermoFisher Scientific). A binary gradient was applied using 0.1% (v/v) formic acid in (A) water and (B) 80% acetonitrile: 0–5 min, 4–4% (B); 5–133 min, 4–32% (B); 133–152 min, 32%–48% (B); 152–155 min, 48–100% (B); 155–170 min, 100–100% (B); 170–171 min, 100–4% (B); 171–180 min, 4–4% (B). The instrument was run in data-dependent mode with 1.8 kV spray voltage, 275°C ion transfer capillary temperature, and the acquisition was performed with the full MS scanned from 700 to 2000 in positive ionization mode. Stepped higher-energy C-trap dissociation (HCD) at 30 ± 10% was applied to obtain tandem MS/MS spectra with m/z values starting from 120.

### Glycoproteomic Data Analysis

Glycopeptide fragmentation spectra were annotated using Byonic software (Protein Metrics, CA, United States) against the reviewed UniProt protein database. Common modifications, including including cysteine carbamidomethyl, methionine oxidation, asparagine deamidation and glutamine deamidation were assigned. The glycan database used for the search were previously published ((Wu et al., 2010; Wu et al., 2011)).

### Molecular Dynamic Simulation of S Protein on ACE2

The 3D structure of S protein and ACE2 complex was obtained from PDB (PDB code 7DF4) (Xu et al., 2021). The most abundant glycans for each ACE2 glycosite were modeled and attached to the protein using CHARMM-GUI((Park et al., 2019)). Additionally, the fully-desialylated glycans were modeled and attached to generate a fully-desialylated homolog of the ACE2 glycoprotein. The models were solvated using the TIP3P water model, and counterions were added to neutralize the system. The CHARMM carbohydrate force field ((Guvench et al., 2011)) and CHARMM36m force field ((Huang et al., 2017)) were used for the carbohydrate and protein structures. Equilibration was performed at 303.15 K over 10 ps. Molecular dynamics simulation was performed using NAMD software package version 2.13 ((Acun et al., 2018)) at 303.15 K under NPT conditions over 5 ns with an output every 10 ps. Long-range electrostatics were evaluated using the particle-mesh Ewald (PME) method ((Mallajosyula et al., 2015)). Covalent bonds involving hydrogen were constrained with the SHAKE algorithm ((Jo et al., 2017)). After dynamics simulations, trajectories were loaded onto VMD for visualization and analysis ((Humphrey et al., 1996)). Specifically, the intermolecular hydrogen-bonding interactions (donor-acceptor distance 3.0 Å, angle cutoff 20°) of each glycan in the fully-sialylated and desialylated forms were compared over the simulation period.

## Results

### Inhibition of Virus Binding by Human Milk Oligosaccharides

**FIGURE 1:**
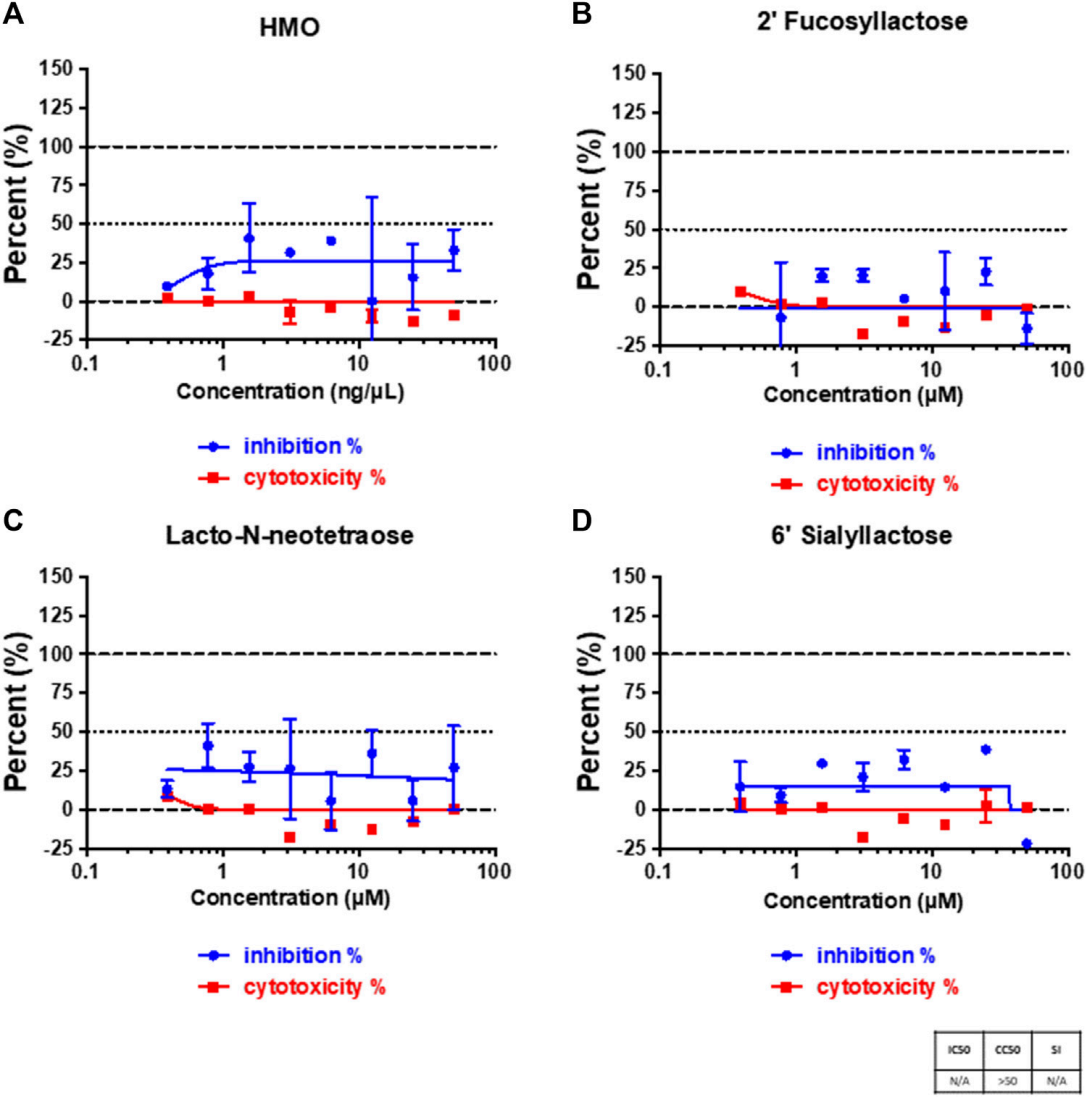
Viral infection on cells and cytotoxicity assays. Cell plates were pre-treated with pooled HMOs **(A)**, 2′-FL **(B)**, LNnT **(C)**, and 6′-SL **(D)**, respectively. The treatment was performed for 2 h at 37°C (5% CO_2_) prior to infection. The percent of infected cells was determined for each well based on GFP expression. All samples were run in triplicate on both an assay plate and a toxicity plate.

Human milk oligosaccharides (HMOs) contain a number of unique structures that can be used to rapidly screen the glycan specificity of the virus. We tested whether SARS-CoV-2 virus could be inhibited by HMOs. We first examined whether pooled samples of purified HMOs from seven different mothers could affect the binding of SARS-CoV-2 virus on Vero E6 cells. Figure 1A showed that the binding capability was affected by the HMO mixture to about 25%. HMOs contain compounds with terminal fucose, sialic acid and galactose. To identify the functional components that could specifically affect binding, we further tested native individual compounds that contained these terminal saccharides. The HMOs 2′-fucosyllactose (2′-FL), 6′- sialyllactose (6′-SL), and lacto-N-neotetraose (LNnT) were selected for this study because they represent many of the structures and are abundant in mothers’ milk. 2′-FL and 6′-SL were produced by adding fucose or a N‐acetylneuraminic acid (Neu5Ac) (Castanys-Muñoz et al., 2013) to the lactose core, respectively. Lacto-N-neotetraose (LNnT) is a neutral HMO with a galactose terminus and contained neither fucose nor sialic acid. The infection studies showed that 2′-FL did not diminish infection, while both 6′-SL and LNnT showed some diminished infection with the latter being slightly more effective than the former and to a similar extent as the pooled sample (Figure 1). However, the variations at the different concentrations are large, particularly for LNnT.

Due to limitations with working on the whole intact virus, we moved the research towards using the S protein as a surrogate for the virus. To validate this model, we performed the experiments on the S protein using the fluorescent labeling and immunofluorescence imaging. SARS-CoV-2 enters host cells *via* the angiotensin-converting enzyme 2 (ACE2) receptor, which binds the receptor binding domain (RBD) of the S protein (12). The Human Protein Atlas (HPA), a website resource for protein expression profiles in cells, tissues and organs (https://www.proteinatlas.org/) (Thul and Lindskog, 2018; Digre and Lindskog, 2021) was used to select the host cell with ACE2 expression. HepG2 was selected after confirming ACE2 expression with labeled antibody and immunofluorescence on the cell membrane (Supplementary Figure S1).

**FIGURE 2:**
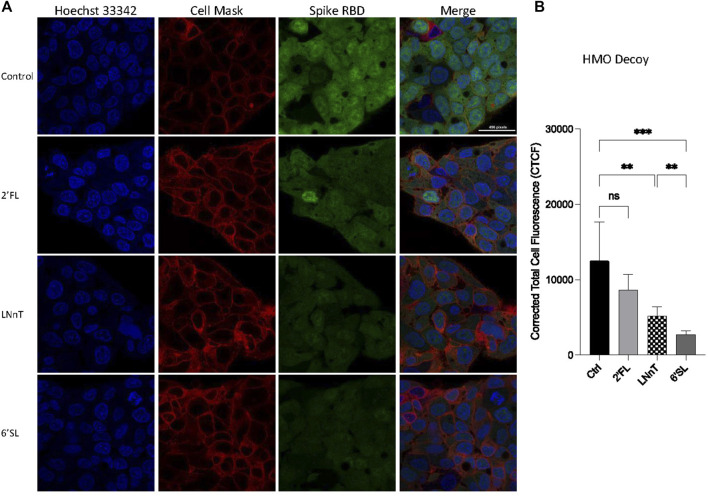
Inhibition of HMOs on the binding between HepG2 cells and Spike protein RBD. Fluorescent labelled proteins were preincubated with 1 mg per ml 2′-FL, 6′-SL, and LNnT, respectively. The preincubation was performed at room temperature for 30 min. **(A)** Immunofluorescence for S protein RBD binding. The columns (from left to right) show staining of nuclear acid (Hoechst 33342), plasma membrane (CellMask™ Deep Red), S protein RBD, and merged image. Scale bar, 496 pixels. **(B)** Quantification of fluorescent intensity of spike protein RBD binding. Fluorescence intensity was quantified for selected cell area. Quantification was performed with software ImageJ. Asterisks indicate the statistical significance between groups compared (**p< 0.01%; ***p< 0.001%; ns *p* < 0.05).

In order to verify further whether HMOs block viral adhesion, we tested the ability of the selected HMO compounds to inhibit RBD binding to HepG2 cells with immunofluorescence. Preincubating HepG2 cells with HMOs did not decrease the binding between the RBD and the cells suggesting that the HMOs did not block binding sites on the host cell surface (Supplementary Figure S2). We then tested whether the HMOs could block or alter the RBD of the virus by preincubating the RBD and the HMOs before introduction to HepG2 cells. Fluorescently labelled RBD was preincubated with 2′-FL, LNnT and 6′-SL separately then allowed to interact with host cells (Figure 2A). Quantitation of fluorescent signal intensity showed that HMOs blocked binding of RBD to cells presumably reflecting the behavior of the intact virus. The RBD was blocked only slightly by 2′FL (not statistically significant), more by LNnT (significant), and the most by 6′SL (Figure 2B). Comparison of LNnT and 6′SL showed that latter one is more effective (significant). The data further showed that HMOs can potentially function as decoys to affect SARS-CoV-2 adherence.

### Determining SARS-CoV-2 Binding Through Variable Glycocalyx Expression

**FIGURE 3:**
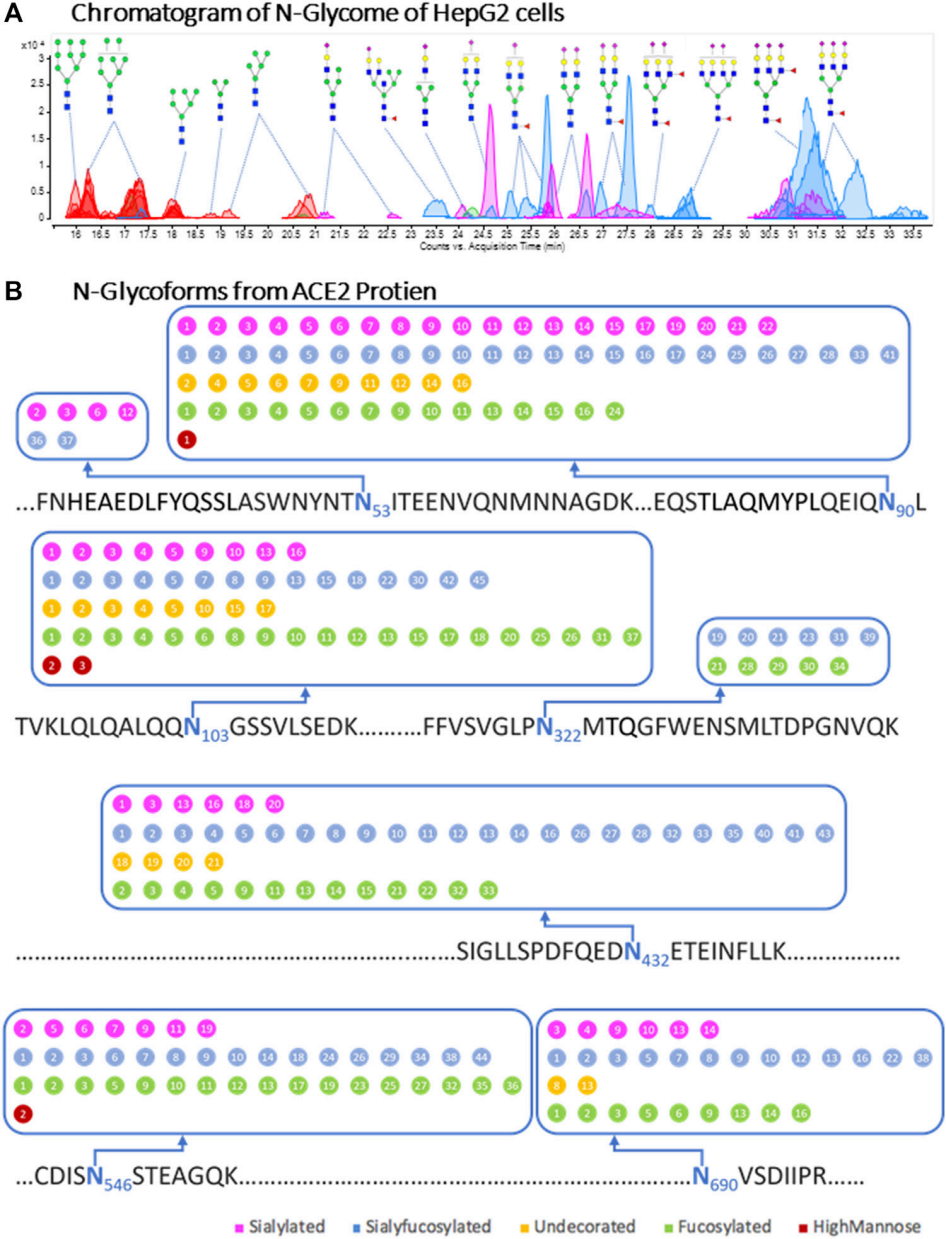
Cell membrane N-glycome and site-specific occupancy of ACE2 receptor in HepG2 cells. **(A)** Individual N-glycan species of HepG2 host cells. LC-MS peaks were color coded to assign glycan subtype. Abundant peaks are annotated with putative structures. Symbol nomenclature is used for representing glycan structures (https://www.ncbi.nlm.nih.gov/glycans/snfg.html). **(B)** Site-specific occupancy of ACE2 receptor in HepG2 cells. The N-glycoforms from ACE2 protien extracted from HepG2 cells are distributed on seven glycosites. The labeled numbers inside dots denote identified individual glycan and the details were shown in Supplementary Table S2.

The notable decrease in binding caused by 6′-SL drew our attention to sialic acids as potential receptors on the cell surface. To further investigate the effects of cell surface glycans on RBD binding, we altered the cell membrane glycans through transferase inhibitors. We first characterized the glycan of the cell membrane and ACE2 on the native cell line. For this analysis, complex and hybrid type glycans were combined to distinguish them from oligomannose type. The N-glycan profile shows a notable abundance of sialylated and sialyfucosylated structures (Figure 3A). The most abundant N-glycan compositions had multiple fucose and sialic acid (N-acetylneuraminic acid or Neu5Ac) residues such as Hex_6_HexNAc_5_Fuc_2_NeuAc_3_, Hex_6_HexNAc_5_Fuc_1_NeuAc_3_ and Hex_5_HexNAc_4_Fuc_1_NeuAc_2_. Glycoproteomic analysis of the cell membrane revealed seven glycosites on the ACE2 protein of HepG2 cells. The *N*-glycoforms of the ACE2 protein extracted from HepG2 cells were diverse and the most common structures were both fucosylated and sialylated (Figure 3B and Supplementary Table S1). For comparison, we analyzed the glycosylation of commercial recombinant ACE2 protein expressed from HEK293 (Supplementary Figure S2 and Supplementary Table S2) and found them to be similar to those expressed by HepG2 (Table 1). Both proteins were highly sialylated and fucosylated with limited amounts of high-mannose glycans.

**TABLE 1:** Summary of Glycoproteomic Profiles of ACE2 proteins.

Subtype of N-Glycans detected on glycosites	Fucosylated	Sialyfucosylated	Undecorated	Sialylated	HM
Recombinant Human ACE2	147	175	68	119	16
ACE2 from HepG2 cells	100	120	38	65	5

The number of glycofmorms was shown in Table 1.

In order to explore the effects of glycocalyx on RBD binding, we metabolically altered the cell surface glycome by treating the cells with inhibitors. To diminish fucosylation on the HepG2 cell surface, we employed a fucosyltransferase inhibitor, 2-fluoro-L-fucose (2F-Fucose). To inhibit sialylation, a sialyltransferase inhibitor 3-fluorinated sialic acid (3-F-Sia) was used. To enrich high-mannose glycans, kifunensine (Kif) was applied to prevent mannose trimming. To determine whether these changes in glycosylation affected ACE2 expression on the cell membrane, we probed the cells with fluorescently labeled antibodies (Supplementary Figure S4). These experiments showed no significant changes in protein expression for ACE2 in any of the glycan modification procedures.

**FIGURE 4:**
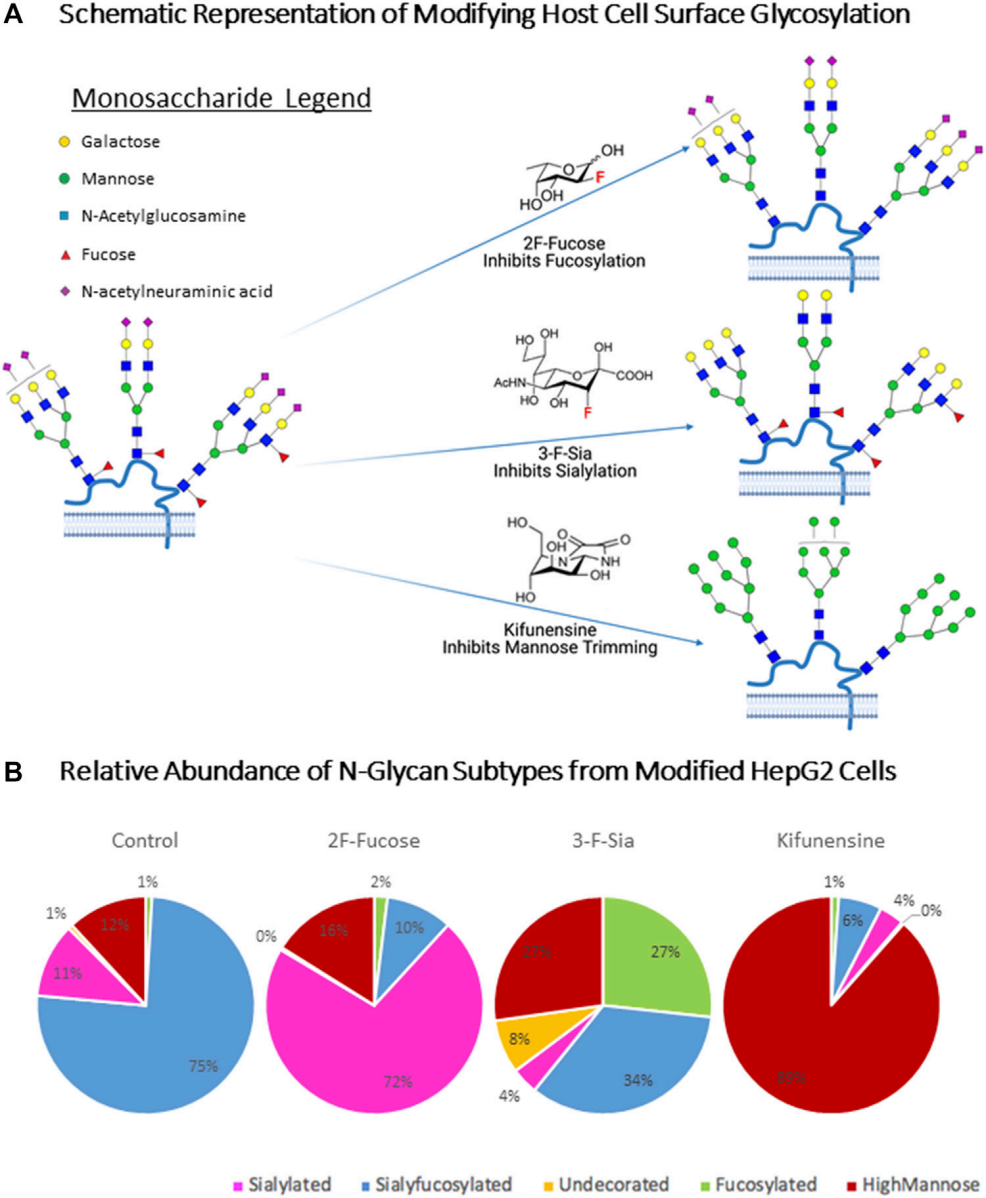
Host Cell Surface Glycome Modification. 2F-Fucose (Fucosyltransferase Inhibitor); 3-F-Sia (Sialyltransferase Inhibitor). **(A)** Metabolic engineering stargey for altering host cell glycosulation. Symbol nomenclature is used for representing glycan structures (https://www.ncbi.nlm.nih.gov/glycans/snfg.html). **(B)** N-Glycome Profiles of unmodified and modified HepG2 cells from LC-MS analysis. Compound list and details are shown in Supplementary Table S3. Pie charts were color coded to assign glycan subtype. Numbers inside pie charts denote the relative abundance of each identified glycan subtype.

The predicted behavior of each substrate are shown in Figure 4A. Compositional profiles were generated for the modified cells, using the sum of the intensities for similar glycan types from the LC-MS analysis. These inhibitors have recently been applied for altering cell surface glycosylation to yield similar results (Zhou et al., 2021) (Figure 4B). 2F-Fucose inhibits fucosylation by being converted to the sugar nucleotide GDP-2F-Fuc ((Villalobos et al., 2015)). It then accumulates in the cell and binds to the transferase and prohibits the enzyme from adding fucose to the nascent chain, thereby decreasing fucose expression on the cell surface (Zhou et al., 2017). The sialyfucosylated N-glycans decreased from 75 to 10% after inhibition with 2F-Fucose treatment. The sialyfucosylated N-glycans were converted to sialylated (only) ones. For example, the abundant sialyfucosylated compound Hex_5_HexNAc_4_Fuc_1_NeuAc decreased (9.6–1.9%, relative abundance) relative to the unfucosylated species Hex_5_HexNAc_4_Fuc_0_NeuAc_2_ which increased in abundance from 3.7 to 19% (Supplementary Table S3). The sialylation pathway was inhibited using 3-F-Sia, a fluorinated sialic acid substrate [cytidine monophosphate (CMP)–SiaFAc]((Suzuki et al., 2015)), which binds more strongly to the enzyme thereby prohibiting the transfer of sialic acids. Treatment with 3-F-Sia decreased the relative abundances of all sialyfucosylated N-glycans from 75 to 34%. Simultaneously, the relative abundance of fucosylated (only) species increased from 1 to 27%. Thus, it appears that the inhibitors are highly effective diminishing fucosylated and sialylated structures, respectively.

**FIGURE 5:**
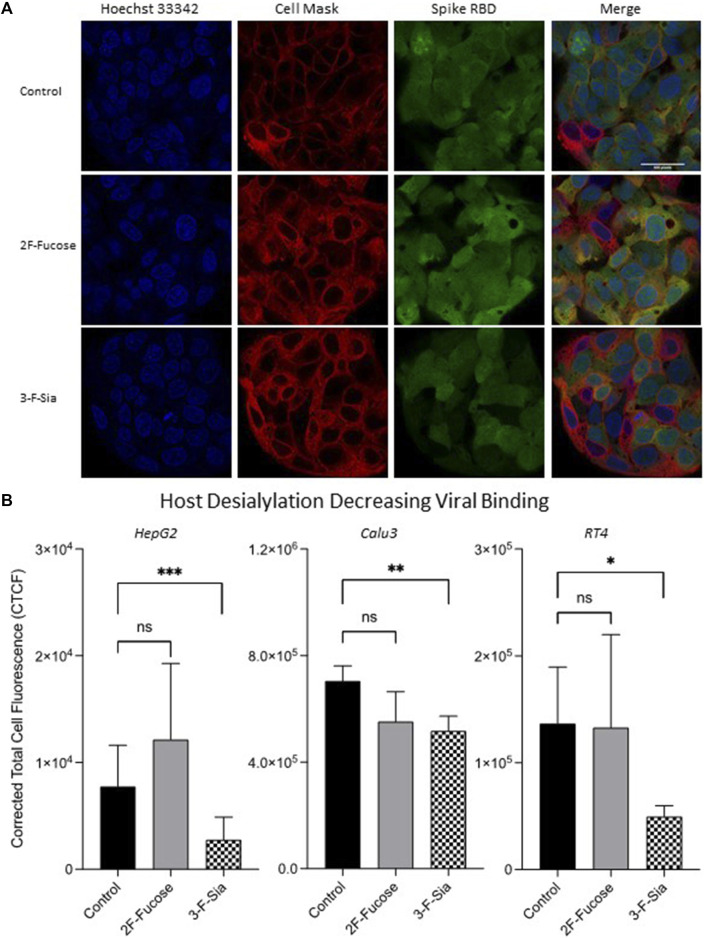
Remodeling host glycome alters binding between host cells and spike protein RBD. 2F-Fucose (Fucosyltransferase Inhibitor); 3-F-Sia (Sialyltransferase Inhibitor). **(A)** Immunofluorescence for S protein RBD binding with modified HepG2 cells. The columns (from left to right) show staining of nuclear acid (Hoechst 33342), plasma membrane (CellMask™ Deep Red), S protein RBD, and merged image. Scale bar, 600 pixels. **(B)** Quantification of fluorescent intensity of spike protein RBD binding. Fluorescence intensity was quantified for selected cell area. Quantification was performed with software ImageJ. Asterisks indicate the statistical significance between groups compared (*p< 0.05%; **p< 0.01%; ***p< 0.001%; ns *p* < 0.05).

After confirming that glycan alteration had taken place in host cells, immunofluorescence analysis was used to observe the effect of host glycome alterations on viral binding. Treatment of 2F-Fucose did not affect RBD binding to the HepG2 cell significantly as observed by immunofluorescence imaging (Figure 5A). However, inhibition of sialylation by 3-F-Sia decreased the S protein RBD binding with HepG2 cells by 64% (Figure 5B), indicating that the binding was likely mediated by sialic acid residues on the host cell surface. Similar trends were observed in other cell lines with ACE2 expression, namely Calu3 and RT4 (Figure 5B). Desialylation inhibited the binding from S protein RBD significantly, and decreased fucosylation did not change the extent of the binding.

**FIGURE 6:**
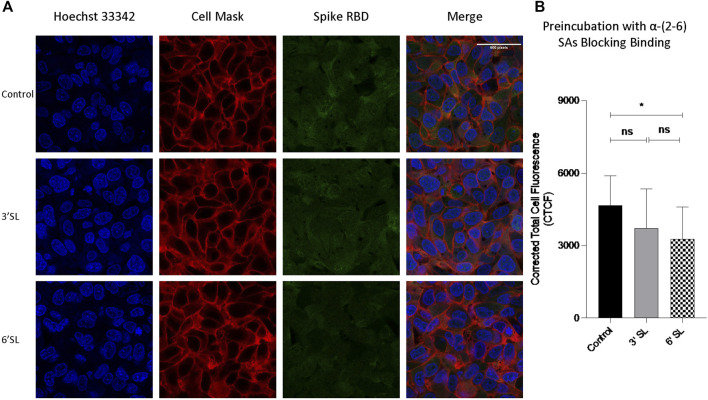
Sialylated HMOs diminished binding of spike protein RBD to HepG2 Cells. Fluorescent labelled proteins were preincubated with 1 mg per ml 3′-FL (3′-sialyllactose) and 6′-SL (6′-sialyllactose) respectively at room temperature. **(A)** Immunofluorescence for S protein RBD binding with cells. The columns (from left to right) show staining of nuclear acid (Hoechst 33342), plasma membrane (CellMask™ Deep Red), S protein RBD, and merged image. Scale bar, 600 pixels. **(B)** Quantification of fluorescent intensity of spike protein RBD binding. Fluorescence intensity was quantified for selected cell area. Quantification was performed with software ImageJ. Asterisks indicate the statistical significance between groups compared (*p< 0.05%; ns *p* < 0.05).

In mammalian cells, terminal sialic acids are commonly found in α-glycosidic linkage to the C-3 or C-6 hydroxyl of galactose *via* α-(2,3)- or α-(2,6)-linkage for N-glycans (Varki A et al., 2015). In nasal mucosa, α-(2-6)-sialic acids are dominant ((Shinya et al., 2006)) with significantly less detected in the lung ((Nicholls et al., 2007)). We further investigated linkage specificities for RBD binding by preincubating the RBD with sialylated HMOs. A significant decrease of RBD intensity was observed after preincubation with 6′‐SL (Figure 6), confirming S protein RBD binds with sialic acids. Comparsion of α-(2-3) with α-(2-6) showed a slight preference against α-(2-6), however the difference was not statistically significant.

Fucosylated glycans were also observed on ACE2 proteins in HepG2 cells (Supplementary Table S1). Terminal α-(1,2) and α-(1,3)-fucose residues are commonly found in mammalian cells ((Ma et al., 2006; Schneider et al., 2017)). To confirm that fucosylation is less important, 2′-FL and 3′-fucosyllactose (3′-FL), components of HMOs, were used ((Ma et al., 2006; Schneider et al., 2017)). Preincubating the S protein RBD with 2′-FL or 3′-FL did not significantly alter binding (Supplementary Figure S5). The S protein RBD again showed little affinity to terminal fucose residues on host cells.

**FIGURE 7:**
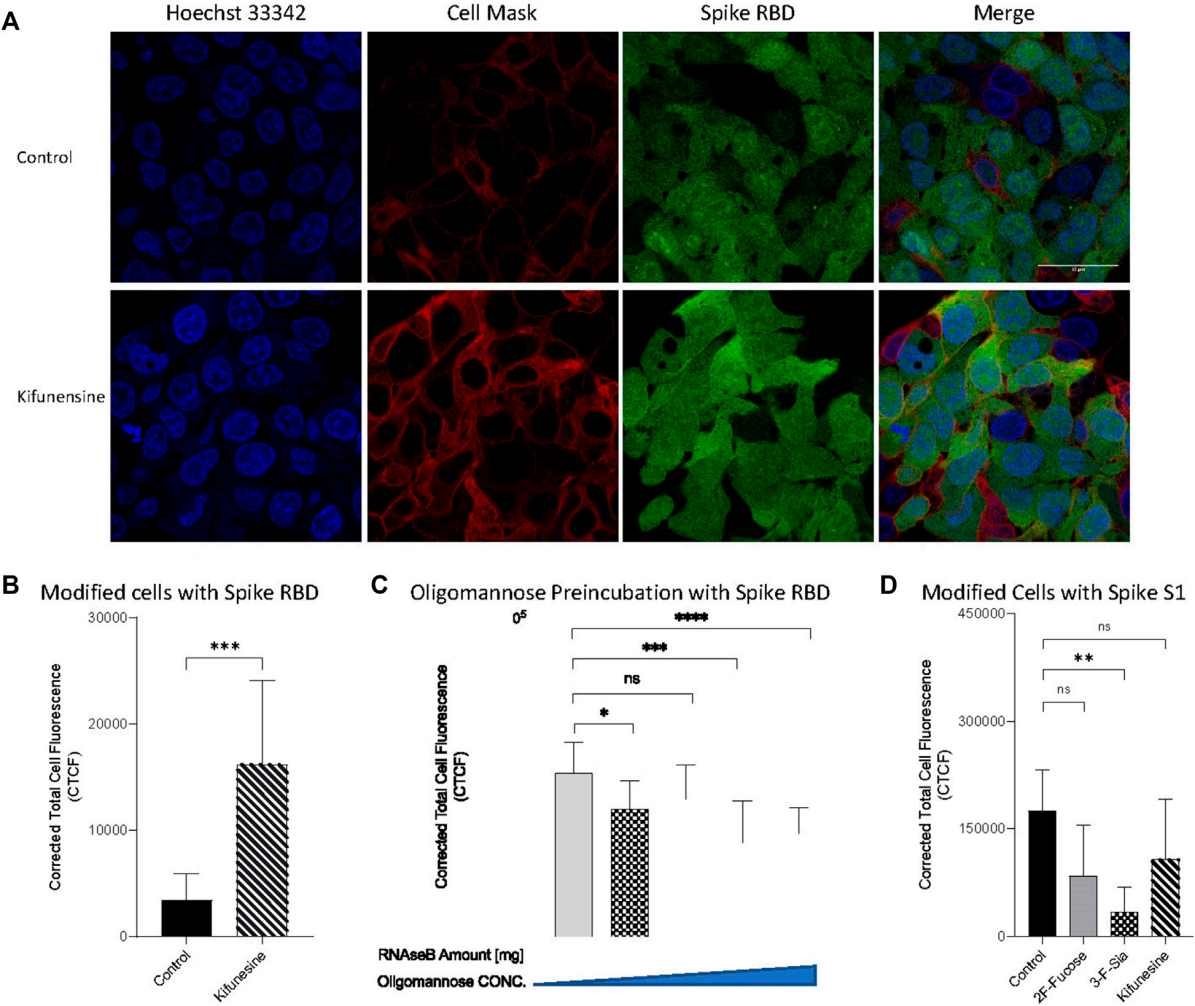
Introducing high mannose glycans into viral binding. **(A)** Immunofluorescence for S protein RBD binding with modified cells. The columns (from left to right) show staining of nuclear acid (Hoechst 33342), plasma membrane (CellMask™ Deep Red), S protein RBD, and merged image. Scale bar, 600 pixels. Quantification of fluorescent intensity of spike protein RBD **(B**,**C)** or S1 subunit **(D)** binding. **(C)** Fluorescent labelled proteins were preincubated with purified high mannose at room temperature for 30 min before binding. Fluorescence intensity was quantified for selected cell area. Quantification was performed with software ImageJ. Asterisks indicate the statistical significance between groups compared (*p< 0.05%; **p< 0.01%; ***p< 0.001%; ****p< 0.0001%; ns *p* < 0.05).

Glycosylation of HepG2 included primarily complex and hybrid type structures with fewer high mannose structures. The latter have been reported as important mediators in host-virus binding for human coronaviruses HKU1((Ma et al., 2006; Schneider et al., 2017)) and severe acute respiratory syndrome (SARS) (Han et al., 2007). We remodeled the cell surface to produce primarily oligomannose and determined its effects on SARS-CoV-2 binding. Kifunensine (Kif) is commonly used to inhibit the α-mannosidase-I ((Kommineni et al., 2019)), thereby preventing mannose trimming to increase oligomannose-type glycans ((Han et al., 2007; Choi et al., 2018)). Our LC/MS data also proved its increasing the relative abundance of oligomannose to 89% in whole cell N-glycome as shown in Figure 4. Introduction of Kif to the cell resulted in a fourfold increase in the binding as measured by immunofluorescence imaging (Figures 7A,B). N-Glycans, released from RNase B, was also employed to examine high mannose type binding. Preincubation with the oligomannose decreased the binding of S protein RBD with host cells (Figure 7C). This effect was dose dependent with higher concentrations preventing binding more strongly. High mannose glycans on host cell surface can therefore increase the adherence of S protein RBD.

To further validate the binding of the spike protein with the host glycocalyx, we used spike protein S1 subunit, a longer polypeptide segment of the S protein and includes the sequence of RBD. Treatment of the cell line with 2F-Fucose did not change the binding between S1 subunit and host cells (Figure 7D). Similarly, the use of 3-F-Sia significant decreased the fluorescent intensity of the assay demonstrating again that the spike protein binds to sialic acids. Surprisingly, the use of Kif in the cell culture no longer increased binding with the S1 subunit. The binding studies showed that there was no significant change in binding relative to the control.

### Molecular Dynamics Calculations of ACE2 and S Protein Interactions

**FIGURE 8:**
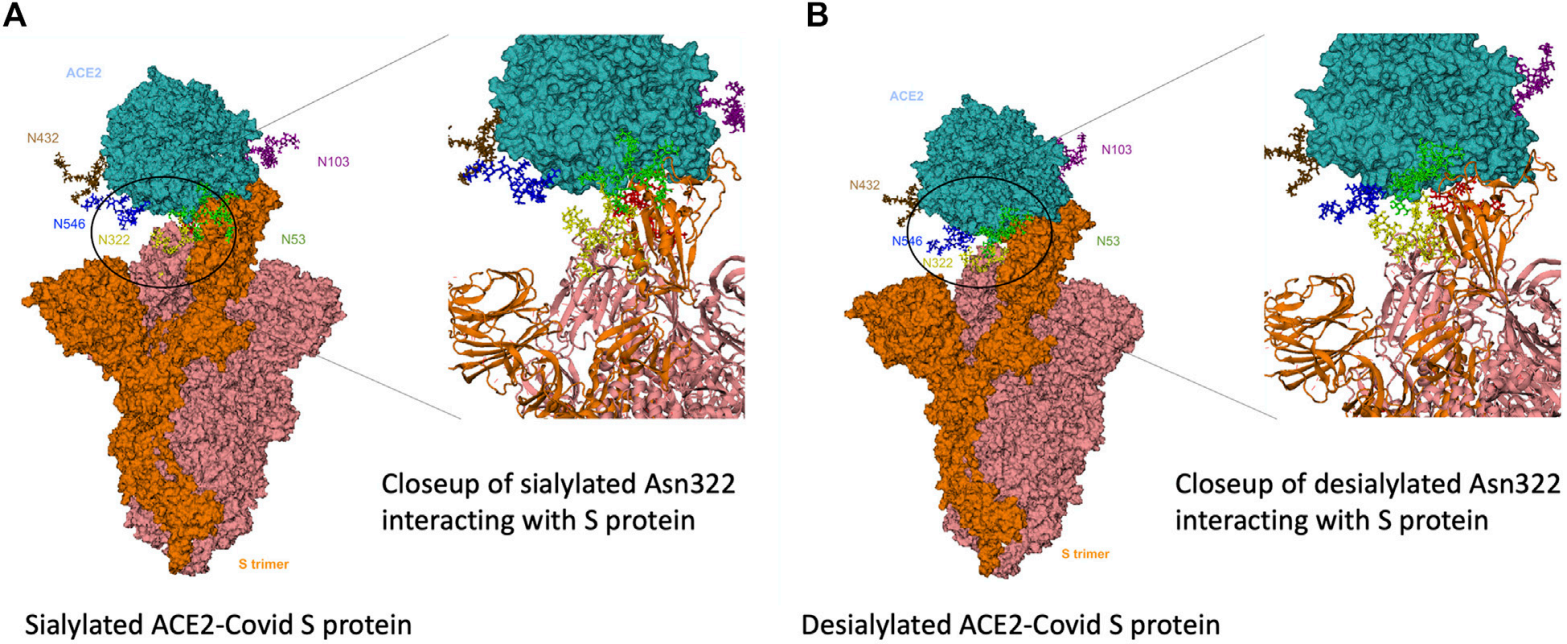
Modelled sialylated and desialylated ACE2-Covid S protein complexes. 3D structural modeling of glycosylated ACE2 interacting with S-protein. Results from glycomics and glycoproteomics of HEPG2 cell lines were used to generate **(A)** fully-desialylated and **(B)** fully-sialylated homologs of ACE2, interacting with S-protein.

**FIGURE 9:**
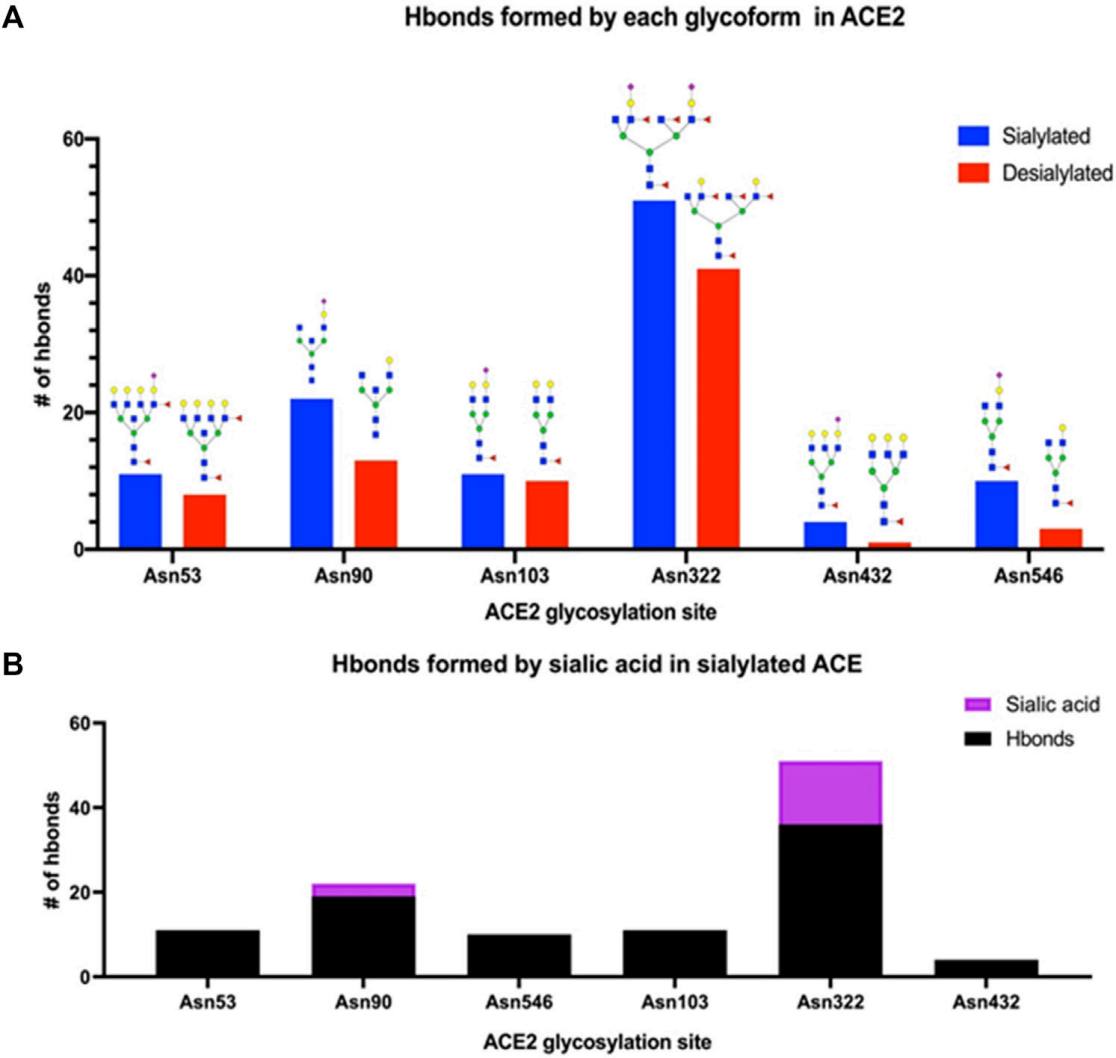
Interactions of glycosylated ACE2 and S-protein were revealed using molecular dynamics simulations. **(A)** The number of intramolecular hydrogen bonds was drastically higher for each fully-sialylated N-glycan compared to the desialylated glycoform. **(B)** For Asn 90 and Asn 322, the sialic acid residue in the glycoform accounted for ∼10% of hydrogen bonds.

To gain further insight into the interactions between the primary receptor ACE2 (Lan et al., 2020; Nguyen et al., 2021; Wong et al., 2004) and the SARS-CoV-2 S protein, we performed molecular dynamics calculations on the interacting complex. Based on the glycoproteomic results for ACE2 from the HepG2 cell line (Supplementary Table S1), we constructed a model with selected glycoforms on ACE2. ACE2 contained seven occupied N-glycan sites corresponding to Asn 53, 90, 103, 322, 432, 546, and 690 (Figure 8A). From the quantitative glycoproteomic results and the Protein Data Bank-derived complex (PDB ID: 7DF4) (Xu et al., 2021), the most abundant glycan at each site were modelled with CHARMM-GUI(32). The resulting structure, shown in the “up” conformation, was selected because it represented the activated complex prior to invasion. Molecular dynamics simulations were performed on the complex with solvent and associated ions for 5 ns (*See* Methods and Materials Section). After the simulations, the number of hydrogen bonds formed between the ACE2 glycans and S protein determined. For comparison, the same calculations were performed on the fully desialylated ACE2 homologs (Figure 8B). The results showed that many of the glycans on ACE2 interacted with the S protein through hydrogen bonding interactions. Comparison of the fully sialylated and desialylated glycans showed significantly lower number of hydrogen bonds (based on 3 Å, donor-acceptor distances) particularly on Asn 90 (22 hydrogen bonds by glycan) and Asn 322 (51 hydrogen bonds by glycan) of the desialylated homolog (Figure 9A). These results are consistent with earlier simulations performed by Zhao et al. on ACE2 - S who noted that both glycan sites were also the most interactive in the complex ((Han et al., 2007; Choi et al., 2018)). Furthermore, when the sialic acids were considered relative to other monosaccharide residues (3 by sialic acid at Asn 90, 15 by sialic acid at Asn 322), their contributions to the overall interactions were proportionally larger (Figure 9B).

## Discussion

Glycans on the host cell membrane and on viral proteins play key roles in the infection of SARS-CoV-2. Viral glycosylation has been the primary focus of glycomic studies related to the virus. Indeed, the spike protein is highly glycosylated with at least 17 N-glycosylation and two O-glycosylation sites identified ((Shajahan et al., 2020; Sanda et al., 2021)). We found two occupied N-glycosites on spike RBD (Supplementary Table S4) consistent with earlier findings. However, the host cells were also highly glycosylated. The LC-MS glycomic profile of HepG2 shows cell membrane with an abundance of high mannose-type glycans as well as complex-type structures with a high degree of sialylation. These structures are also branched with a combination of bi, tri, and higher antennary structures. The HepG2 cell lines was selected for its expression of ACE2, and these highly sialylated branched structures were similarly present in the protein further alluding to the importance of sialylation in at least the host-virus adhesion process.

The results showed that sialic acid in human milk oligosaccharides (HMOs) can block the binding of virus on the cell membrane. These results are further supported by recent findings that show similar deflecting properties of sialylated HMOs toward the S protein of SARS-CoV-2 ((Han et al., 2007; Choi et al., 2018)) and illustrating further the protective nature of human milk against these pathogens. HMOs are more similar to O-glycans in structure, however N-glycans on membrane proteins similarly provide sialic acid on their termini. Altering the glycans on the cell membrane, while maintaining the expression levels of proteins such as ACE2, further shows that sialic acid on the cell surface induces stronger binding to the virus. ACE2 is itself highly sialylated, in the cell line used in this study and from commercial sources (mainly from HEK293). ACE2 expressed recombinantly in other cell lines have similar glycosylation profiles that are similarly rich in sialylation. Deeper structural analysis showed that the binding prefers a specific linkage, namely α-(2,6)-sialic acids. Interestingly, the human influenza virus has a similar preference for binding ((Trebbien et al., 2011; Wu et al., 2015; Sieben et al., 2020)). Perhaps not coincidently, human epithelial in the nasal mucosa is rich in α-(2,6)-sialic acid, which is also more abundant than the isomer α-(2,3)-sialic acid, the binding site of avian bird flu ((Shajahan et al., 2020; Sanda et al., 2021)).

The binding of sialic acid point to specific protective measures by the host. In breast fed infants, HMOs can provide some protection. Human milk is also full of proteins that are highly sialylated such as the immunoglobulins and lactoferrins ((Almond et al., 2013; Smilowitz et al., 2013; Jorgensen et al., 2017; Morniroli et al., 2021)). In adults, pathogen deflection is performed by the mucus layer. SARS-CoV-2 is a respiratory disease reaching deep into the respiratory tract and the lungs. It also infects the intestine (Wang et al., 2020), with both types of tissues protected by a mucus layer constructed around high molecular weight glycoproteins called mucins ((Hansson, 2019)). Mucin are expressed in epithelial surfaces of gastrointestinal, genitourinary, and respiratory tracts, where they also shield the surface against chemical and physical damages ((Trebbien et al., 2011; Wu et al., 2015; Sieben et al., 2020)). While mucins are covered primarily by O-glycans that are similar to human milk oligosaccharides, they contain the same sialic acid termini as N-glycans. The mucus layer therefore presents a myriad of potential binding sites for commensal and pathogenic microbes ((Tran and Ten Hagen, 2013; Tailford et al., 2015)), and shedding mucins is a defense strategy against pathogen infection.

The high mannose glycans were also strongly bound in the shorter version (RBD) of the S protein. However, in the longer homolog (S1 subunit) this binding was diminished. These results suggest that that there is a high mannose binding site on the S protein that is potentially shielded in the longer homolog. On the other hand, high mannose glycans are typically not found on epithelial cells ((Tran and Ten Hagen, 2013; Tailford et al., 2015)) and are not abundant in the blood. However, they are much more abundant in the tissue samples compared to serum. These glycans are found in cancer cells (Radcliffe et al., 2007; Möginger et al., 2018) and stem cells ((An et al., 2012)). The levels of several oligomannose type glycans are upregulated in tumor tissue ((Balog et al., 2012; Ruhaak et al., 2015)). The role of mannose residue as a host receptor has been studied and proved in the microbe-host interactions, such as Salmonella enterica subsp. enterica serovar typhimurium (S. t*yphimurium*) (Park et al., 2016), influenza virus ((Reading et al., 2000; Upham et al., 2010)), dengue virus ((Miller et al., 2008)) and human immunodeficiency virus (HIV) (Nguyen and Hildreth, 2003). Mannan is usually employed for studying mannose binding with virus ((Han et al., 2007; Upham et al., 2010; Hulswit et al., 2019; Routhu et al., 2019)). The mannans are highly heterogeneous in length and branching. The repeating α-(1,6)-linked mannose backbone is usually branched by short chains of α-(1-2) and α-(1-3)-linked mannose structures ((Varki et al., 2015)). In this study, we used oligomannose released form RNase B ((Roberts et al., 2000)) instead of mannan. The released high mannose glycans were determined with mass spectrometry (Supplementary Figure S6), and all those structures have been found in human cell glycomes.

The integrated method developed here, which includes alteration of cell surface glycan products through specific inhibitors, coupled with the enrichment of the membrane proteins and extensive glycomic and glycoproteomic analysis provides a new platform for obtaining structural specificity in host-microbe interactions. Glycans are common targets for many commensals and pathogens alike. This method will have great utility in identifying glycan targets of individual microbes and even toxins that bind glycans. The method is made possible by recent advancements in novel glycosyl transferase inhibitors that produce specifically glycosylated membrane proteins. We noted that the conversion to a glycan type is never fully complete. There are residual endogenous glycans due to the differences in turnover of different glycoproteoform ((Wong et al., 2020)). However, the ability to perform glycomic profiling with LC-MS provides a guiding assay to examine the extent of the glycomic transformation.

## Conclusion

The study supports a mechanism for binding of SARS-CoV-2 to the cell membrane that is primarily mediated by glycans. The preferred target of the S protein is sialylated glycans with α-(2,6)-sialic acids on the termini positions. The virus likely binds to cells and tissues rich in sialylated glycans, whether N-, O-, and potentially even glycolipids that are found in the surface of the epithelial surface. The airway epithelial is rich in sialic acids and in particularly α-(2,6)-sialic acids. In this regard, the human influenza virus and SARS-CoV-2 have the same binding preference in the host membranes. Invasion of SARS-CoV-2 likely occurs when the virus fortuitously binds to the ACE2 protein, which itself is highly sialylated. The alignment between the S and the ACE2 protein is further facilitated by hydrogen binding interactions between the sialylated glycans of the host cell and the polypeptide of the S protein.

## Data Availability

The data presented in the study are deposited in the MassIVE data repository, accession number MSV000088722.
