# Supplementary figures and images for: Host Cell Glycocalyx Remodeling Reveals SARS-CoV-2 Spike Protein Glycomic Binding Sites
Source: Front Mol Biosci. 2022 Mar 14;9:799703. doi: 10.3389/fmolb.2022.799703 (PMC8964299; doi:10.3389/fmolb.2022.799703)

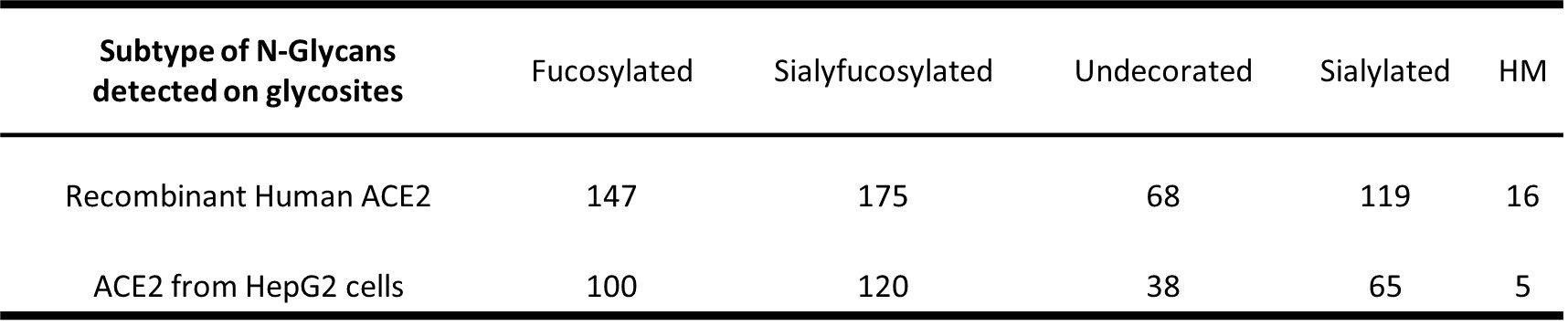

Supplement: Supplementary file 2 [file Image1.JPEG]
